# Epithelial–mesenchymal transition in cancer

**DOI:** 10.1002/1878-0261.12094

**Published:** 2017-07-04

**Authors:** Aristidis Moustakas, Antonio Garcia de Herreros

**Affiliations:** ^1^ Department of Medical Biochemistry and Microbiology Science for Life Laboratory Biomedical Center Ludwig Institute for Cancer Research Uppsala University Sweden; ^2^ Cancer Research Program Institut Hospital del Mar d'Investigacions Mèdiques Barcelona Spain; ^3^ Departament de Ciències Experimentals i de la Salut Universitat Pompeu Fabra Barcelona Spain

The generation of an organized architectural pattern during tissue formation is a process of paramount importance during development and organogenesis. A large proportion of the vertebrate body is made of epithelial tissues; these provide organized barriers between organ compartments and also differentiate into glandular, secretory specializations (Kahata *et al*., 2017). One feature of epithelial developmental history is their ability to generate mesenchymal cell types via a process best known as epithelial–mesenchymal transition (EMT) (Hay, 1995; Lim and Thiery, 2012). The barrier function of epithelia is also met in the organization of the vascular and lymphatic walls that are built by endothelial and lymphatic endothelial cells, respectively (van Meeteren and ten Dijke, 2012). A similar generation of mesenchymal cells from the endothelium is also relevant in heart morphogenesis. Actually, one of the very early observations of the EMT (or more properly, of the endothelial–mesenchymal transition (van Meeteren and ten Dijke, 2012) stems from studies of endothelial cells and formation of tissue diversifications within the heart, such as the valves and their septa.

The EMT does not necessarily generate terminally differentiated mesenchymal cells but rather produces intermediate cell phenotypes that retain the potential to regenerate new epithelial tissue via the process of mesenchymal–epithelial transition (MET) (Nieto *et al*., 2016). EMT and MET may represent distinct and interdependent biological processes or, alternatively, they may embody reversible phases of one and the same process. As the locations where an EMT and a connected MET take place within the vertebrate body are often distinct, evidence for the reversibility of these two processes is often ambiguous, and the process is of unclear biological relevance. Clear cases of the MET can be understood by studying the dedifferentiation of mesenchymal cells, for example, fibroblasts, to renal epithelium during nephrogenesis (Davies, 1996). Moreover, MET is required for the generation of pluripotent stem cells from fibroblasts using the popular Yamanaka factor protocols (Sanchez Alvarado and Yamanaka, 2014). Sequential EMT and MET processes are needed to differentiate, for example, induced pluripotent cells to hepatocytes (Li *et al*., 2017). These studies indicate the relevance of these transitions in embryonic development and can provide new hints on their mechanisms.

EMT and MET are not only embryonic physiological processes, but become activated during chronic inflammation, wound healing, and cancer metastasis (Lambert *et al*., 2017; Nieto *et al*., 2016). In the latter case, a substantial body of literature describes the contribution of EMT to the invasive state of various carcinomas (epithelial tumors), whereas MET is thought to operate once metastatic cells have reached a distant site of secondary growth (Nieto *et al*., 2016). In this respect, it is difficult to demonstrate and even understand how the two processes connect with each other when they are separated in time and space, and the cells that connect the EMT to MET may undergo multiple pathophysiological and epigenetic changes in the interim period. In addition, specific *in vivo* murine models of cancer metastasis occasionally dispute the contribution of the EMT to metastatic spread away from the primary oncogenic site (Fischer *et al*., 2015; Zheng *et al*., 2015). Further complication to the above concept is the proposal that cancer‐associated EMT is not complete, and epithelial tumor cells generate intermediate phenotypes that express mixed epithelial and mesenchymal genes; such ‘hybrid’ cells may exert more malignant properties compared to the more differentiated epithelial or mesenchymal cells (Jolly *et al*., 2016).

Although the EMT field has progressed to generate long lists of proteins and noncoding RNA, whose identity both marks and functionally defines the process, all modern studies focus on phenotypic analyses of key regulatory processes that can be referred to as hallmarks of the EMT (Nieto *et al*., 2016). These include disruption of cell–cell adhesion complexes, the most characteristic of which involve the adherens, tight, and desmosomal junctions; remodeling of the three classes of cytoskeleton, microfilaments, microtubules, and intermediate filaments; and finally, the transformation of the extracellular matrix and associated cell surface receptors, which signifies a change in cell–matrix adhesion and type of cellular motility. These hallmarks of EMT functionally have been linked to processes of cell motility, local destruction of the basement membrane that aligns the epithelia, and the generation of chemoresistant tumor cells with stem cell abilities that contribute to cancer recurrence after therapy (Nieto *et al*., 2016).

The cellular adaptations characterizing the EMT hallmarks are driven by growth factor signaling pathways, such as transforming growth factor β (TGF‐β), Wnt, fibroblast growth factor (FGF), and Notch (Kahata *et al*., 2017; Lamouille *et al*., 2014). These pathways instruct the expression and activity of several transcription factors, called EMT transcription factors (EMT‐TFs), which operate in coordination with changes in the chromatin of several genes (epigenetic remodeling) in order to promote the EMT (Nieto *et al*., 2016; Tam and Weinberg, 2013). The signaling networks that connect growth factors, their receptors, intracellular signaling mediators, and the actions of the EMT‐TFs, leading to regulation of expression of genes involved in cell–cell junction, cell–matrix junction, and cytoskeletal remodeling have been reviewed exhaustively over the past years (Lamouille *et al*., 2014). For this reason, the current issue of *Molecular Oncology* does not focus on this exciting aspect of tumor cell biology. It is worth, however, to stress the fact that the complexity of the signaling networks driving the EMT and MET is ever increasing as new regulators of key components of these networks; for example, enzymes controlling the stability of EMT‐TFs (Díaz and de Herreros, 2016) are gradually uncovered.

Important areas that remain open to investigation relate to the translation of the basic knowledge on EMT on various aspects of oncology. Are the EMT and MET truly important for cancer progression and metastasis or are they, as some studies have suggested (Fischer *et al*., 2015; Zheng *et al*., 2015), dispensable? How complete is tumoral EMT? Thus, does EMT produce cells with mesenchymal traits or only provide epithelial cells with a more plastic phenotype? Which types of tumors do require an EMT to invade? Do they have differences in other cancer traits (i.e., resistance to apoptosis) relative to tumors invading through collective migration? Does MET occurring in metastatic sites represent a reverse or essentially distinct and independent process relative to the EMT that initiated invasiveness in the primary tumor? Do the processes studied in normal stem cells, and their generation by dedifferentiation technologies, help us understand the link between carcinomas and the generation of disseminating cancer stem cells that maintain both a potential for resistance to drug therapy and a metastatic ability, as analyzed by populations of circulating tumor cells? As gene regulation, cell–cell, and cell–matrix contacts are abundant in all cell types, and as the EMT‐TFs are expressed in various cell compartments, are EMT‐TFs relevant to the biology of nonepithelial cells in the tumor microenvironment (for instance, activated fibroblasts)? Are EMT‐TFs also relevant to the molecular oncology of nonepithelial tumors? At the time when high‐throughput molecular technologies allow the quantitative measurement of mRNA, microRNA, protein, and metabolite abundance in tumors, can small quantitative changes in molecular concentration ever find relevant application to the oncology clinic and guide a new phase of cancer pathology? Can the vast knowledge in the differentiation changes collectively happening during the EMT and MET generate applications in the area of cancer diagnosis? As therapy improvement is the primary aim of cancer research, can the lessons provided by the complexity and the plasticity incorporated in the concept of EMT generate new avenues for therapeutic intervention? In particular, our contemporary anticancer arsenal evolves more and more toward the combinatorial use of ‘smart drugs’ that attack multiple molecular pathways and that guarantee limited chance to disease relapse. Operating under this framework for future cancer therapy development, how can the EMT paradigm enrich the targets of combinatorial anticancer therapy and suggest new pipelines for clinical trials, with emphasis on metastatic disease? These are the questions that experts in each respective area have attempted to provide their up‐to‐date views.

By combining the expertise of founders of the EMT field with younger investigators, this compendium aims at leading the research front and illuminating both scientists and the wider public that are interested in fundamental problems of oncology. Studies on EMT provide exciting clues about the evolution and adaptations that human tumors undergo through their history, a history that represents the awakening of embryonic developmental scenarios taking place in adult bodies over the trajectory of unpredictable genetic and environmental insults that characterize normal human life.
